# Side-by-side plastic stent insertion for refractory post-endoscopic sphincterotomy bleeding after covered metal stent placement

**DOI:** 10.1055/a-2780-8661

**Published:** 2026-02-09

**Authors:** Takehiko Koga, Naoaki Tsuchiya, Yusuke Ishida, Makoto Fukuyama, Keisuke Matsumoto, Yi-Ling Ko, Fumihito Hirai

**Affiliations:** 1 38208 Department of Gastroenterology and Medicine, Fukuoka University Faculty of Medicine, Fukuoka, Japan

Post-endoscopic sphincterotomy (ES) bleeding is a common adverse event following endoscopic retrograde cholangiopancreatography (ERCP 1 ). The placement of a covered self-expandable metal stent (CSEMS) has been reported as an effective rescue hemostatic technique by providing mechanical compression at the duodenal papilla 2 3 . However, bleeding can occasionally persist despite the CSEMS placement 4 5 . Herein, we report a novel rescue hemostatic method for such challenging cases ( Video 1 ).

Rescue hemostasis with side-by-side plastic stent insertion for post-endoscopic sphincterotomy bleeding refractory to covered metal stent placement.Video 1

**Fig. 1:**
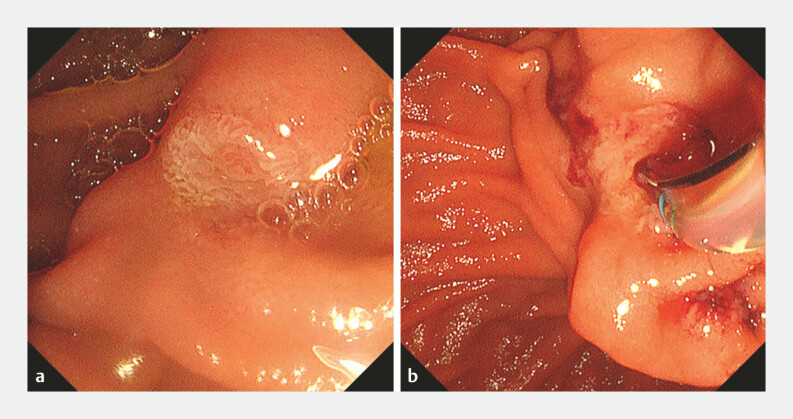
Endoscopic images before endoscopic sphincterotomy. **a** The duodenal papilla before endoscopic sphincterotomy. **b** Sphincterotome positioned at the papillary orifice just before incision.

**Fig. 2:**
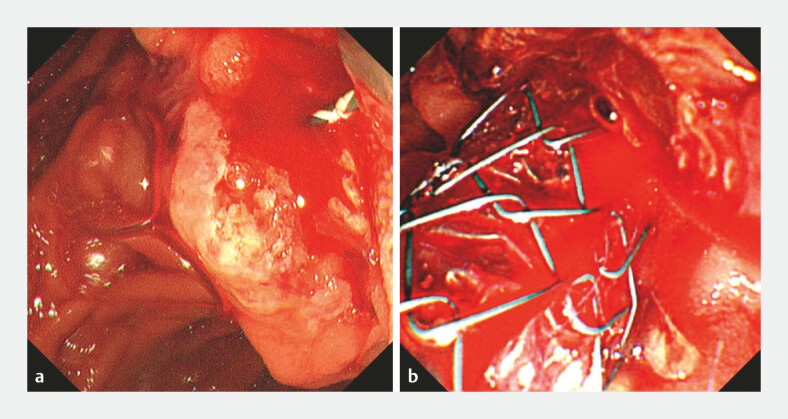
Endoscopic images after endoscopic sphincterotomy. **a** Active bleeding from the duodenal papilla after endoscopic sphincterotomy. **b** Persistent bleeding despite the placement of a covered self-expandable metal stent.

**Fig. 3:**
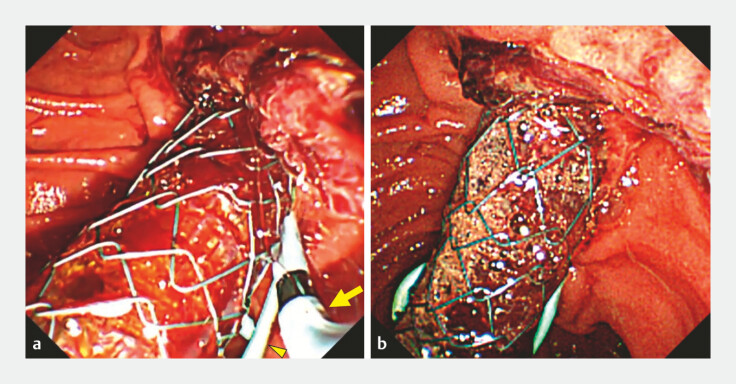
Endoscopic images after additional plastic stent placement. **a** An endoscopic view showing the side-by-side placement of a plastic stent (arrow) beside the covered self-expandable metal stent, following the placement of a pancreatic plastic stent (arrowhead). **b** Hemostasis achieved after additional plastic stent placement.

An 86-year-old woman with a history of Billroth I reconstruction underwent ERCP for distal biliary obstruction. After cholangiography, ES was performed ( Fig. 1 ), and continuous bleeding from the duodenal papilla occurred thereafter ( Fig. 2 **a** ). Initial endoscopic hemostasis using a balloon tamponade and hemostatic gel failed. A 5-Fr pancreatic plastic stent was placed to prevent post-ERCP pancreatitis, followed by deployment of a CSEMS (HANAROSTENT, 10 mm × 10 cm; M.I. Tech Co., Seoul, Korea). However, profuse bleeding persisted, and the source was unclear ( Fig. 2 **b** ). Subsequently, a 7-Fr plastic stent (ADFlap Stent, 14 cm; SILUX, Saitama, Japan) was inserted side-by-side with the CSEMS, providing additional compression to the duodenal papilla and achieving hemostasis ( Fig. 3 ). No rebleeding or adverse events occurred.

**Fig. 4:**
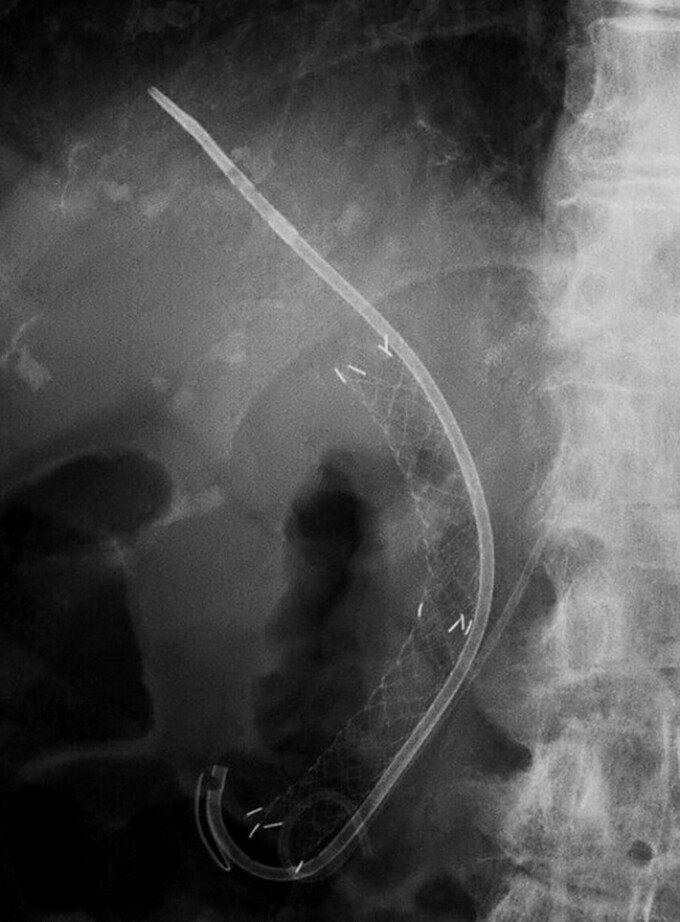
A fluoroscopic image after the procedure. A fluoroscopic view showing the side-by-side placement of covered self-expandable metal and plastic stents.

In this case, the additional plastic stent was positioned on the anal side of the CSEMS, opposite to the sphincterotomy incision ( Fig. 4 ). This likely altered the angle and tension of the CSEMS, enhancing direct mechanical compression at the bleeding site. This simple modification may serve as a practical rescue technique when the CSEMS placement alone is insufficient, expanding its hemostatic potential in challenging post-ES bleeding.

Endoscopy_UCTN_Code_CPL_1AK_2AC
